# Pharmacokinetics of acetaminophen after a single Oral administration of 20 or 40 mg/kg to 7–9 Day-old foals

**DOI:** 10.3389/fvets.2023.1198940

**Published:** 2023-07-06

**Authors:** Jenifer R. Gold, Tamara Grubb, Michael H. Court, Nicolas F. Villarino

**Affiliations:** ^1^Wisconsin Equine Clinic and Hospital, Oconomowoc, WI, United States; ^2^Department of Veterinary Clinical Sciences, Washington State University, Pullman, WA, United States

**Keywords:** equine, anti-inflammatory, neonate, plasma disposition, horse

## Abstract

**Background:**

Acetaminophen is utilized in human infants for pain management and fever. Neonatal foals might benefit from administration of acetaminophen but effective and safe dosage regimens for neonatal foals remains to be determined.

**Objective:**

The objective was to determine the plasma pharmacokinetics of acetaminophen following oral administration of a single dose of 20 mg/kg or 40 mg/kg to neonatal foals. A secondary objective was to evaluate any changes in hematology and biochemistry profiles.

**Study design:**

Randomized study.

**Methods:**

Eight clinically healthy 7–9-day old Quarter Horse foals (3 colts and 5 fillies) received a single oral dose of acetaminophen either 20 (*n* = 4) or 40 (*n* = 4) mg/kg. Hematology and biochemistry profiles were evaluated before and 7 days after drug administration. Blood samples were collected before and 8 times after acetaminophen administration for 48 h to quantify plasma acetaminophen concentrations. Plasma pharmacokinetic parameters were estimated using non- compartmental analysis.

**Results:**

The median peak plasma concentrations (and range) occurred at 1.5 (0.5–2) hours, and 1.0 (1–2) hours for the 20 and 40 mg/kg doses. The maximum plasma concentration (and range) was 12 (7.9–17.4) μg/mL for the 20 mg/kg dose and 14 (11–18) μg/mL for 40 mg/kg dose. The median AUC_0-∞_ ranged from 46 to 100 and 79 to 160 h*-μg/mL for the 20 and 40 mg/kg dose, respectively. Hematology and biochemistry profiles remained within normal limits.

**Conclusion:**

Plasma disposition of acetaminophen after oral administration of 20 and 40 mg/kg to neonates is comparable to adult horses. However, safety and the optimal dosage regimen of acetaminophen for treating pain and or pyrexia in neonates in this age group remains to be determined.

## 1. Introduction

Acetaminophen is a common over-the-counter analgesic and antipyretic drug used in human medicine (1). In human patients, acetaminophen can be administered orally, intravenously or per rectum and can provide analgesia within 40 min with maximal effect at 1 h (1, 2). Aside from being used alone, it has been used in humans in multimodal pain management (3–5).

Acetaminophen is metabolized extensively by the liver. The main metabolic pathways are glucuronidation and sulfation, which in human adult’s accounts for 55 and 30% of acetaminophen metabolism, respectively (6–8). A small fraction (2–5%) of the absorbed dose is excreted unchanged in the urine (8–12). Human neonates have a slower total clearance of acetaminophen with higher sulfation and lower glucuronidation per kg of bodyweight compared to adults (12–16).

Several studies have shown gastric emptying is the rate-limiting the step in the adult horses’ absorptive process of acetaminophen (17, 18). Acetaminophen in adult horses is then rapidly absorbed in the proximal small intestine via passive diffusion (19, 20). In regard to liver function in the equine foal, microsomal enzyme activity increases rapidly by 3–4 weeks of age (21–25) while conjugation takes longer to reach adult levels (24, 25) With respect to acetaminophen in the foal, acetaminophen appears to have linear disposition following a single dose of 10–40 mg/kg in foals (26). However, in adult horses, with repeated dosing of acetaminophen, the disposition is no longer linear, with a decrease in elimination (27–29) suggesting that the disposition of acetaminophen may be age dependent in horses.

In a recent study in 1–3-month-old foals receiving a single dose of acetaminophen, the maximum concentration (Cmax) median and range at the 10 mg/kg dose was 4.4 μg/mL (1.8–5.1) at the 20 mg/kg dose was 6.3 μg/mL (2.6–12.6) and at the 40 mg/kg dose was 14 μg/mL (7.3–18) (26). Further studies are needed to understand the plasma disposition of acetaminophen in neonatal foals.

Acetaminophen can cause dose-dependent liver disease in humans (30, 31). In a study performed on adult horses, acetaminophen administered at a dose of 25 mg/kg orally every 12 h for 30 days caused no detectable renal or hepatic effects as shown by biochemistry profiles (32, 33). Chronically lame adult horses receiving 30 mg/kg orally every 12 h for 21 days had no adverse effects in their liver biopsy histopathology or biochemistry profiles either (28). In foals (1–3-month-old) treated with 10, 20 and 40 mg/kg once orally, no clinically significant changes were noted in the hematology or serum biochemistry parameters, but this is only after a single dose administration (26). Any potential effect of acetaminophen on hematology or serum biochemistry parameters in neonatal foals remains to be determined.

The main objective of this study was to determine the plasma pharmacokinetics of acetaminophen following oral administration of a single dose of 20 mg/kg or 40 mg/kg to neonatal foals. A second objective of this study was to observe any changes in the hematology or biochemistry profiles after oral administration of 20 and 40 mg/kg of acetaminophen.

## 2. Materials and methods

This study was approved by the Utah State University Institutional Animal Care and Use Committee ASAF #11023. Eight clinically healthy Quarter Horses foals (3 colts and 5 fillies) from Utah State University Animal Sciences Department were studied using a randomized study design where 4 foals received 20 mg/kg and 4 foals received 40 mg/kg of acetaminophen once orally, where the foals were selected for 2 different doses by a random generator.^1^ The number of foals was selected to obtain the maximum information from the smallest number of animals. Toxicity studies are conducted on small groups of 3–5 rodents of each sex per dose (34, 35). The non-rodent species groups typically utilize 4–6 animals, hence the reason for selected 4 foals per group (34, 35).

The mean (±standard deviation) body weight (kg) of the foals was 51.0 ± 8.0 kg. All the foals were between 7–9 days of age. All foals were housed with their dams in stalls with an outdoor run, which is the typical management scheme of mare and foals on this property. The foals were allowed to nurse without restrictions during the research project. The mares were fed while the project was ongoing in the mornings and evenings.

### 2.1. Prior to start of the study

The afternoon before the first administration of acetaminophen a Jorvet^2^ extended-use catheter was placed aseptically in one jugular vein of each foal. The catheters were heparin-locked (2.5 mls normal saline,^3^ 2.5 mls of 100 units/mL heparin^4^) until the next morning. The catheters were wrapped with roll gauze^5^ and elastikon tape^6^ to protect the catheter. Whole blood and plasma were obtained the same afternoon for the initial hematology^7^ and biochemistry profile.^8^ These were analyzed at the Utah State Diagnostic Laboratory for confirmation of normal hematology parameters; white cell count (WBC), red cell count (RBC), hemoglobin (Hb), fibrinogen, and total protein (TP), and normal biochemistry parameters including blood urea nitrogen (BUN), creatinine (CR), sorbitol dehydrogenase (SDH), gamma-glutamyl transferase (GGT), and alkaline phosphatase (ALP), triglycerides, aspartate aminotransferase (AST), total bilirubin, and total protein (TP). These biochemistry values were chosen to observe liver and renal values during the study period.

### 2.2. Study time frame

Physical examinations were performed on each foal before drug administration, at the start of the study and three times a day throughout the study.^9^ The examinations included the determination of heart rate, respiratory rate, body temperature, mucous membrane color, capillary refill time and gastrointestinal borborygmi, and palpation of joints and umbilicus. The 500 mg acetaminophen extra strength regular release tablets^10^ were ground in a coffee grinder. The dose of acetaminophen administered was rounded to the closet number of whole tablets based on the weight of each foal in each treatment group (Example-a 48 kg foal getting a 40 mg/kg dose would need 1920 mg of acetaminophen, the actual dose the foal got was 2000 mg or 41.6 mg/kg) Table 1. The ground acetaminophen was mixed with water (24 mls) and karo syrup or molasses (1 mL) and administered orally via a catheter tip syringe (water, karo syrup or molasses and ground acetaminophen).

**Table 1:** Foal weight, acetaminophen dose, dose administered, approximate dose received.

Foal #	Foal weight kg	Acetaminophen dose	Acetaminophen dose received
1	48	40 mg/kg – 1920 mg	2000 mg, dose ≅ 41.6 mg/kg
2	41	20 mg/kg – 820 mg	1,000 mg, dose ≅ 24.4 mg/kg
3	56.8	20 mg/kg – 1,136 mg	1,100 mg dose ≅ 19.8 mg/kg
4	43.2	40 mg/kg – 1728 mg	1800 mg, dose ≅ 41.5 mg/kg
5	65.5	40 mg/kg – 2,620 mg	2,500 mg, dose ≅ 39.0 mg/kg
6	48.6	20 mg/kg – 972 mg	1,000 mg, dose ≅ 20.6 mg/kg
7	49.5	20 mg/kg – 990 mg	1,000 mg, dose ≅ 20.2 mg/kg
8	56.3	40 mg/kg – 2,252 mg	2,250 mg, dose ≅ 40.0 mg/kg

Five mls of blood were withdrawn at each time point from the jugular catheter, after which an additional 5 mls of blood was withdrawn and placed into a plastic heparin blood collection tube. The initial 5 mls of blood was reinjected into the jugular vein through the catheter and the catheters were flushed with normal saline solution. To limit the amount of heparin administered, the catheters were flushed with saline twice a day and twice a day with (50 USP units of heparin/5 mls saline)^11^ to maintain catheter patency. Blood was collected for the pharmacokinetic analysis once before the administration of acetaminophen (time 0) and a total of 8 times 0.50, 1, 2, 4, 8,16, 24 and 48 h post administration of acetaminophen. Blood was also obtained from each foal for hematology and biochemistry profiles which were performed prior to acetaminophen administration and 7 days after the dosage of acetaminophen. The samples were placed on ice until they were spun down. The heparinized samples were centrifuged for the plasma acetaminophen concentrations at 1800 x g for 5 min and the plasma was removed and stored at -80^o^ C until analysis.

The assays were performed within 6 months of collection. A preliminary study using horse plasma samples from our prior study (26) stored under the same conditions showed good stability of acetaminophen concentrations (105 ± 6% of the original value) more than 12 months after the original assay. This is consistent with stability studies of acetaminophen, acetaminophen glucuronide and acetaminophen sulfate in humans’ plasma, which showed minimal change after 6 months of storage at −80^0^ C (36).

### 2.3. Drug quantification and analytical method

Acetaminophen concentrations were measured in the plasma samples by high-performance liquid chromatography (HPLC) with mass spectrometry detection using the same method previously validated for horse plasma [26]. Standard curves were linear (R^2^ > 0.99) over assayed range (0.05–50 μg/mL). The lower and upper limit of quantification was 0.05 μg/mL and 50 μg/mL, respectively.

Assay precision (coefficient of variation) and accuracy (percent deviation from nominal) was evaluated in each run using quality control samples consisting of blank horse plasma spiked with multiple concentrations of acetaminophen. Assay precision was 5, 5, and 11% for 50, 2.5, and 0.05 μg/mL concentrations. Assay accuracy was −6%, +4% and + 16% for 50, 2.5, and 0.05 μg/mL concentrations.

### 2.4. Estimation of plasma pharmacokinetic parameters

Non-compartmental analysis was used to calculate pharmacokinetic parameters (37) as implemented by Phoenix WinNonlin® v8.0.^12^ Estimated pharmacokinetic parameters include; area under the plasma concentration-time curve from 0 h to infinity after dosing (AUC_0-∞_), area under the plasma concentration-time curve from 0 h to the last sampling time (AUC_0-last_), maximum concentration (Cmax), time to maximum concentration (Tmax), half-life of terminal portion of the curve after oral administration.

### 2.5. Data analysis

The effect of acetaminophen treatment on hematology and biochemistry profile parameters were assessed statistically using the Wilcoxon matched-pairs signed-rank test. Dose proportionality was evaluated by comparing the dose-normalized AUC_0-∞_ and dose-normalized Cmax values between dose levels. All statistical comparisons were made using GraphPad Prism v7.4 for Windows.^13^ The level of significance was set at *p* < 0.05.

## 3. Results

The hematology profiles remained within the normal reference range (35, 36). The biochemistry parameters were normal in all but 3 foals. The GGT concentrations were outside the reference range before and after administration of acetaminophen in 1 foal, and alkaline phosphatase concentrations were outside the reference range in 1 foal before acetaminophen administration but were normal post acetaminophen administration. One foal had SDH concentration outside the reference range prior to acetaminophen concentration but was normal 7 days post administration. However, no statistical significance was found (Tables 2, 3). Physical examination parameters also remained within normal limits for all foals throughout the study.

**Table 2:** Hematology parameters (mean and range) from 7–9-day-old foals before and after single oral administration of acetaminophen at 20 mg/kg (*n* = 4) and 40 mg/kg (*n* = 4).

Dosage Regimen	PCV%	White cell X 10^3^/μL	Red cell X 10^6^/μL	Haemoglobin mmoL/L	Plasma protein g/I	Fibrinogen g/I
20 mg/kg pre-dose	36.5 (32–45)	9.3 (5.7–11.2)	9.4 (7.5–10.6)	8.2 (7.1–9.5)	54 (51–60)	1.75 (0–3.00)
20 mg/kg post-dose	35.7 (30–43)	11.7 (8.5–16.5)	9.4 (8.7–11.2)	7.8(6.4–9.7)	57 (51–65)	2.75 (1.00–5.00)
40 mg/kg pre-dose	42 (38–48)	8.6 7.3–13.0	10.2 (7.6–11.2)	9.2 (8.7–9.9)	57 (44–68)	1.71 (0–4.00)
40 mg/kg Post-dose	37.2 (34–41)	9.4 (4.3–13.9)	9.81 (8.7–11.2)	8.6 (8.5–9.12)	56 (44–68)	2.75 (0–5.00)
Reference range*	31–44	7.2–13.5	7.8–10.6	7.3–10.9	51–75	1.60–4.18

There were no statistical differences in any of the parameters for any dose level. *Harvey, J.W., Asquith, R.L., McNulty, P.K., Kivepelto, J., Bauer, J.E. (1984). Haematology of foals up to 1 year old. Eq Vet J 16, 347–353. Axon, J.E., Palmer J.E. (2008). Clinical pathology of the foal. Vet Clin Equine. 24, 357–385. Faramarzi, B., Rich, L. (2019). Haematologic profile in foals during the first year of life. Vet Record 184, 1–5.

**Table 3:** Biochemistry profile (mean and range) from 7–9-day-old foals before and 7 days after a single oral administration of acetaminophen at 20 mg/kg (*n* = 4), and 40 mg/k (*n* = 4).

Dosage Regimen	BUN mmol/L	Creatinine μmol/L	ALP IU/L	GGT IU/L	SDH IU/L	AST IU/L	TBil μmol/L	Triglyceride mmol/L
20 mg/kg Pre-dose	1.1 (0.7–1.4)	81 (79.5–88.4)	1,122 (835–1,457)	43.2 (22–73)	7.8 6.4–9.5	214.5 (201–241)	31.6 (26–37)	1.0 (0.7–1.8)
20 mg/kg Post-dose	1.2 (1.0–1.4)	88.4 (88.4)	1,101 (451–1,551)	48.5 (31–71)	5.2 3.4–7.9	254 (184–309)	27.4 (24–33)	0.81 (0.5–1.1)
40 mg/kg Pre-dose	1.9 (1.0–2.5)	106.1 (106–114.9)	2,347 (1397–3,683)	106 (19–208)	18 6.4–24.9	301 (198–340)	42.7 (31–56)	1.1 (0.85–1.4)
40 mg/kg Post-dose	2.2 (1.4–3.6)	106.1 (97.3–123.8)	1,222 (838–1,632)	138 (24–354)	4.2 1.1–9.8	290 (149–430)	34.2 (19–46)	0.68 (0.27–0.97)
Reference Range*	1.1–5.0	88.4-150.31	861–2,671	14–164	0.1–23.0	146–340	27–85	0.05–1.74

There were no statistical differences in any of the parameters for any dose level.

BUN, blood urea nitrogen; ALP, alkaline phosphatase; GGT, gamma glutamyl-transferase; SDH, sodium dehydrogenase; AST, aspartate aminotransferase; TBILI, total bilirubin.

* Axon, J.E., Palmer J.E. (2008). Clinical pathology of the foal. *Vet Clin Equine.* 24, 357–385. Bauer, J.E., Harvey, J.W., Asquith, R.L., McNulty, P.K, Kivipelto, J. (1984). Clinical chemistry reference values of foals during the first year of life. *Eq Vet J.* 16(4), 361–363. Barton, M.H., LeRoy, B.E. (2007). Bile acid concentrations in healthy and clinically ill neonatal foals*. J Vet Intern Med.* 21, 508–513.Waldridge, B.M. (2013). Review of Serum chemistry interpretation in Neonatal Foals *AAEP Proceedings* 59, 498–500.

**Figure 1:**
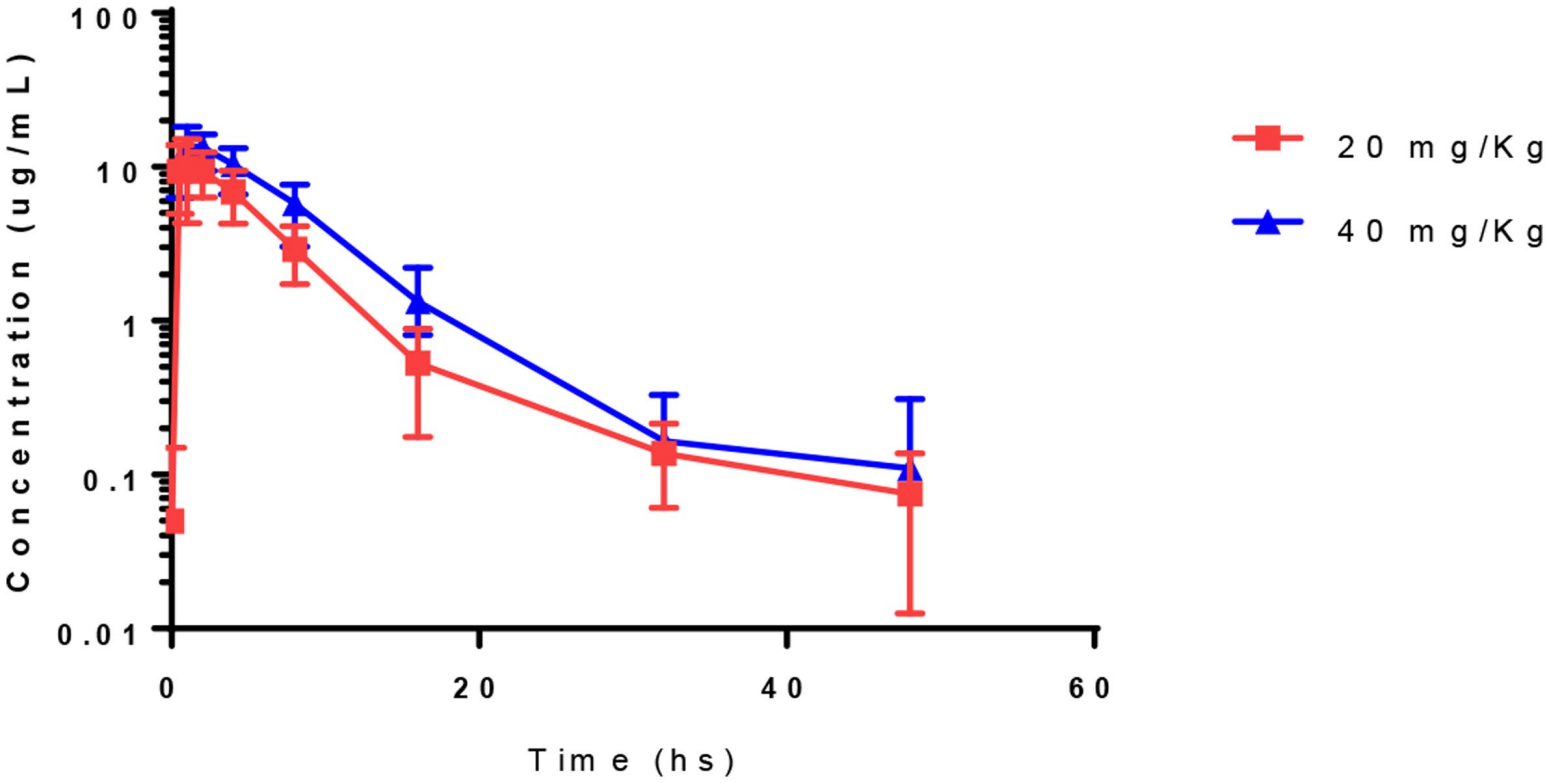
Plasma concentrations of acetaminophen in 7–9 days old foals after a single oral administration at 20 and 40 mg/Kg of body weight (Mean ± SD).

Pharmacokinetic parameters determined by non-compartment analysis are presented in Table 4. Acetaminophen was detected in the plasma of all foals (Figure 1). Peak plasma concentrations occurred from 0.5 to 2 h and 1 to 2 h for 20 and 40 mg/kg and maximum plasma concentration ranged from 7.9–17 μg/mL and 11 to 18 μg/mL for 20 and 40 mg/kg dose level, respectively. AUC_0-∞_ ranged from 46 to 100 h*μg/mL and 79 to 160 h*μg /mL for the 20 and 40 mg/kg dose, respectively. The dose-normalized AUC_0-last_ ranged from 2.2 to 5 and 2 to 4 for the 20 and 40 mg/kg doses, respectively. The dose-normalized Cmax ranged from 0.4–0.9 and 0.3 to 0.5 for the 20 and 40 mg/kg doses, respectively.

**Table 4:** Plasma pharmacokinetic parameters (median (range)) of acetaminophen estimated by non-compartmental analysis in 7–9 days-old foals after oral administration at 20 (*n* = 4 foals) and 40 mg/Kg (*n* = 4 foals) of body weight.

PK parameter	Unit	20 mg/Kg (*n* = 4)	40 mg/Kg (*n* = 4)
HL_Lambda_z	hr	9 (6–14)	6 (4–7)
Tmax	hr	1 0.5 (0.5–2)	1 (1–2)
Cmax	μg/mL	12 (7.9–17.4)	14 (11–18)
AUC_last_	hr*μg/mL	75 (44–99)	121 (78–160)
AUC_INF_obs_	hr*μg/mL	76 (46–100)	123 (79–160)
AUC_last/_dose	N/a	3.8 (2.2–5)	3 (2–4)
AUC0-inf % extrapolated	%	0.7–2.2	0.06–1.7
Cmax/dose	N/a	0.6 (0.4–0.9)	0.4 (0.3–0.5)

AUC_INF_obs_ = area under the plasma concentration-time curve from 0 h to infinity after dosing; AUC_last_ = area under the plasma concentration-time curve from 0 h to last sample time; Cmax = maximum concentration; Tmax = time to maximum concentration. HL_Lambda_z = half-life for the terminal phase. The extrapolated area was less than 5%. K10_HL = Half-life of the terminal portion of the curve after oral administration. The median dose-normalized AUC_0-∞_ and Cmax were not different (*p* > 0.05; Kruskal-Wallis test).

## 4. Discussion

This is the first study to describe the plasma pharmacokinetics and effect on physical examination, hematology, and biochemical profiles of a single oral dose administration of acetaminophen in 7–9-day-old foals. No adverse reactions were observed in any foal treated with acetaminophen. After a single acetaminophen dose administration, the clinical, hematology, and biochemical profiles remained within the reference range except in 3 foals where one foal had elevations in GGT before and after administration of acetaminophen and 1 foal had an elevated alkaline phosphatase level prior to acetaminophen concentration and one foal had a mild elevation of SDH prior to acetaminophen but was normal 7 days post acetaminophen administration. This may be a normal variation with age since the elevations were noted in the foals prior to the administration of acetaminophen and concentrations were lower but still above the reference range 1 week later in the foal with elevated GGT. The safety profile of acetaminophen in neonatal foals needs to be assessed after repeated dosing of acetaminophen to determine if the long-term administration causes liver damage or other adverse effects.

The plasma disposition of acetaminophen in 7–9-day old neonates is comparable to that in older foals (26). However, a relevant difference in the disposition of acetaminophen between neonates and 1–3-months old foals is that the median terminal half-life was relatively longer in neonatal foals (Table 4). The median terminal half-life (range) in the neonatal foals was 9 (6–14 h) and 6 (4–7) hrs for 20 mg/kg and 40 mg/kg, respectively. This finding is different compared to 1–3-month-old foals where the terminal half-life for a single oral dose at 10 mg/kg, 20 mg/kg and 40 mg/kg (range) was 2.6 (1.4–3.4) hrs, 2.8 (2.05–3.9) hrs and 2.7(2.4–7.4) hrs, respectively.

The AUC /dose at 20 mg/kg (range) was larger at 3.8 (2.2–5) in the 7–9 day-old- foals compared to the same dose 1-3-month-foals which was 2 (1.1–3.7), and at 40 mg/kg in 7-9-old-foals was 3 (2–4) compared to the 1-3-month-foals at 40 mg/kg which was 2.6 (2.1–3.6). This discrepancy is likely attributable to the slower drug clearance of acetaminophen in neonates than in older foals. Neonatal foals have an immature biotransformation system which can explain the slower drug elimination of acetaminophen. Neonatal foals have longer half-lives for some drugs versus older foals, particularly between birth and 5 days of age (26, 27). However, foals seem to develop microsomal-mediated metabolic pathways rapidly in the first 3–4 weeks of life and reach the activity of adults by 6–12 weeks of life (26, 27). Differences in the maturity of enzymatic systems involved in the biotransformation of acetaminophen could explain the interindividual variability in neonates and age-dependent differences in the disposition of this drug. The clinical relevance of the age-dependent disposition of acetaminophen in foals deserves further research.

No evidence for dose non-proportionality in AUC_0-last_ occurred in this study and this agrees with the results of a previous study in 1–3 months old foals treated with acetaminophen at 20 and 40 mg/kg orally once (26). In contrast, the Cmax in the 7-9-day-old foals did not increase in a dose-proportional manner, as the median Cmax of acetaminophen increased from 12 μg/mL to 14 μg/mL, for the 20 and 40 mg/kg dose levels in comparison to the 1-3-month old foals where the median Cmax at 10 mg/kg was 4.4 μg/mL, at 20 mg/kg was 6.3 μg/mL and 40 mg/kg was 14 μg/mL which increased in a dose proportional fashion. It is possible, that this discrepancy between the two cohort of foals is result of age-depended differences in rate and extent of distribution of acetaminophen. A larger study is necessary to confirm these dose proportionality findings. Furthermore, it is not possible to determine if this finding would be clinically relevant as the therapeutic concentration of acetaminophen remains to be determined in the horse.

The effective serum concentration of acetaminophen that elicits 50% of the maximum anti-pyretic drug response (EC_50_) in adult humans has been estimated to be between 15.2 and 16.5 μg/mL and the minimum therapeutic concentration for pyrexia is established to be 10 μg/mL (7–14). In human neonates, effective concentrations for pyrexia have been established to be 10–11 μg/mL in different studies (38–41). In children that have undergone tonsillectomy, 10 μg/mL was found to alleviate pain, but lower strength may be sufficient with less pain (42). In the present study, the Cmax was higher than 10 μg/mL in all foals treated with 40 mg/kg of acetaminophen. If the human analgesic serum acetaminophen concentration is extrapolated to foals, the administration of 40 mg/Kg of acetaminophen may result in analgesic concentrations, assuming that the maximal plasma concentration of acetaminophen is positively correlated with the drug effect. Two studies in horses have looked at the pharmacodynamics of analgesia in horses (28, 33). However, more studies are needed to determine the optimal dosage regimen for treating pain and pyrexia.

This study generated novel information but has several limitations. One limitation of this study is the number of foals was small, with only 4 foals in each group. Thus, the finding should be interpreted with care and knowledge that further studies are needed before dosing foals of any age.

The main limitation of this project is that the study design does not allow assessment of acetaminophen safety in with multiple doses because the drug was administered once and because the biochemistry and hematology was assessed 7 days after the administration, which may have served to capture only delayed or long-lasting effects. Considering the significant liver toxicity concern in humans, and futures studies should be designed specifically to rule out liver adverse effects of acetaminophen in foals.

Liver toxicity is a significant finding in human studies at elevated doses (30, 31) but has not been noted in multi-dose administration in horses. (27–29, 32, 33) Since neonatal foals have rapidly changing liver function, impairment due to liver toxicity might change the metabolism, prolong the half-life, and alter drug disposition (1, 2, 12–14, 21–25). Thus, further studies are needed to assess safety of multiple dosing of acetaminophen in neonatal and older foals.

In conclusion, this study is the first to describe the disposition of acetaminophen in neonatal foals at 20 mg/kg and 40 mg/kg doses and the resulting pharmacokinetic parameters. The results of this study are encouraging for the potential to use of acetaminophen in foals. Further studies are needed to assess the safety, analgesic, and antipyretic effects and the disposition of acetaminophen after repeated administration of different doses in foals of varying ages.

## Data availability statement

The original contributions presented in the study are included in the article/supplementary material, further inquiries can be directed to the corresponding author.

## Ethics statement

The animal study was reviewed and approved by the Study protocols were approved via Institutional Animal Care and Use Committee IACUC Approval ASAF# 11023.

## Author contributions

JG wrote the grant and assisted for the project, abstract, and manuscript. TG helped the manuscript and data collection. MC performed the lab HPLC analysis of samples and assisted the manuscript. NV performed the pharmcokinetic analysis, statistical analysis, and assisted the manuscript. All authors contributed to the article and approved the submitted version.

## Funding

This work was supported by the WSU Intramural Funding-Luella Gottstein Endowment for Equine Research, Stanley L. Alder Research Fund.

## Conflict of interest

The authors declare that the research was conducted in the absence of any commercial or financial relationships that could be construed as a potential conflict of interest.

## Publisher’s note

All claims expressed in this article are solely those of the authors and do not necessarily represent those of their affiliated organizations, or those of the publisher, the editors and the reviewers. Any product that may be evaluated in this article, or claim that may be made by its manufacturer, is not guaranteed or endorsed by the publisher.

## Footnotes

^1^Random.org, Dublin. Ireland.

^2^Jorvet labs, Loveland, CO. USA.

^3^Sargent Pharmaceuticals, Schaumberg, IL. USA.

^4^Medex Supply, Passiac, NJ. USA.

^5^Becton, Dickenson and Company, Franklin Lakes, NJ. USA.

^6^Johnson and Johnson, New Brunswick, NJ. USA.

^7^Siemens Advia 120, Siemens Health Care Diagnostics Inc., Tarrytown, NY. USA.

^8^Siemens Dimension Xpand Plus, Siemens Health Care Diagnostics Inc., Tarrytown, NY. USA.

^9^Better Living Brands, Pleasanton, CA. USA.

^10^Sigma Aldrich, St Louis, MO. USA.

^11^Toronto Research Chemicals, Toronto, Ontario, Canada.

^12^Certara Princeton, NJ. USA.

^13^GraphPad Software, San Diego, CA. USA.
